# A Comprehensive Evaluation of Dioxins and Furans Occurrence in River Sediments from a Secondary Steel Recycling Craft Village in Northern Vietnam

**DOI:** 10.3390/molecules29081788

**Published:** 2024-04-15

**Authors:** Hung Xuan Nguyen, Xuyen Thi Nguyen, Hang Thi Hong Mai, Huong Thi Nguyen, Nam Duc Vu, Thao Thi Phuong Pham, Trung Quang Nguyen, Dat Tien Nguyen, Nam Thanh Duong, Anh Le Tuan Hoang, Tung Ngoc Nguyen, Nhan Van Le, Ha Viet Dao, Minh Truong Ngoc, Minh Quang Bui

**Affiliations:** 1 Faculty of Chemistry, Graduate University of Science and Technology, Vietnam Academy of Science and Technology, 18 Hoang Quoc Viet Street, Cau Giay, Hanoi 11353, Vietnam; xuanhung450809@yahoo.com (H.X.N.); nguyenthixuyenk57a@gmail.com (X.T.N.); 2 Center for High Technology Research and Development, Vietnam Academy of Science and Technology, 18 Hoang Quoc Viet Street, Cau Giay, Hanoi 11353, Vietnam; maithihonghang_sdh21@hus.edu.vn (H.T.H.M.); nguyenthihuongtb79@gmail.com (H.T.N.); namvd@yahoo.com (N.D.V.); thaopham284@gmail.com (T.T.P.P.); ngtiend@gmail.com (D.T.N.); namduongthanh@gmail.com (N.T.D.); hoangletuananh.cretech@gmail.com (A.L.T.H.); tungnguyen.vast@gmail.com (T.N.N.); levannhan.na@gmail.com (N.V.L.); anphuminh1011@gmail.com (M.T.N.); 3 Faculty of Chemistry, Hanoi University of Science, Vietnam National University, Hanoi, 19 Le Thanh Tong Street, Hoan Kiem, Hanoi 11021, Vietnam; 4 Institute of Environmental Science and Public Health, 18 Hoang Quoc Viet Street, Cau Giay, Hanoi 11353, Vietnam; nqtrung79@gmail.com; 5 Institute of Oceanography, 1 Cau Da Street, Nha Trang 57111, Khanh Hoa Province, Vietnam; daovietha69@gmail.com

**Keywords:** dioxin/furan contaminants, HRGC/HRMS, craft village, steel recycling

## Abstract

This first study investigated the presence of dioxins and furans in river sediments around a craft village in Vietnam, focusing on Secondary Steel Recycling. Sediment samples were collected from various locations along the riverbed near the Da Hoi Secondary Steel Recycling village in Bac Ninh province. The analysis was conducted using a HRGC/HRMS-DFS device, detecting a total of 17 dioxin/furan isomers in all samples, with an average total concentration of 288.86 ng/kg d.w. The concentrations of dioxin/furan congeners showed minimal variation among sediment samples, ranging from 253.9 to 344.2 ng/kg d.w. The predominant compounds in the dioxin group were OCDD, while in the furan group, they were 1,2,3,4,6,7,8-HpCDF and OCDF. The chlorine content in the molecule appeared to be closely related to the concentration of dioxins and their percentage distribution. However, the levels of furan isomers did not vary significantly. The distribution of these compounds was not dependent on the flow direction, as they were mainly found in solid waste and are not water-soluble. Although the hepta and octa congeners had high concentrations, when converted to TEQ values, the tetra and penta groups (for dioxins) and the penta and hexa groups (for furans) contributed more to toxicity. Furthermore, the source of dioxins in sediments at Da Hoi does not only originate from steel recycling production activities but also from other combustion sites. The average total toxicity was 10.92 ng TEQ/kg d.w, ranging from 4.99 to 17.88 ng TEQ/kg d.w, which did not exceed the threshold specified in QCVN 43:2017/BTNMT, the National Technical Regulation on Sediment Quality. Nonetheless, these levels are still concerning. The presence of these toxic substances not only impacts aquatic organisms in the sampled water environment but also poses potential health risks to residents living nearby.

## 1. Introduction

Craft villages (CVs) are small production models that have been developed for a long time and play a role in Vietnam economics. However, the activities of CVs still cause serious environmental pollution [1]. Bac Ninh is the province with the most traditional CVs in Northern Vietnam. Among them, Da Hoi (DH) is well known to a CVs, causing serious environmental pollution that is difficult to treat.

DH, at around 400 years old, is known to contain one of the largest CVs of Secondary Steel Recycling (SSR) in Northern Vietnam, with approximately 1700 production facilities engaging in activities such as steel casting, rolling, plating, nail making, and steel mesh weaving, with a daily output of nearly 1000 tons of iron and steel products. Here, the steel casting, rolling, and plating furnaces operate around the clock. In many places, thick black smoke is emitted through ducts, blending with the pungent smell of asphalt, coal dust, metal dust, iron filings, and the odors of chemicals, rotting water, and waste [2].

Every day, DH CVs use about 40,000 tons of various types of coal and 18,000 cubic meters of water, discharging 150 tons of industrial waste (including coal slag, scrap metal, and iron filings) and less than 1 ton of domestic waste, along with approximately 15,000 cubic meters of wastewater. All of this waste is discharged directly into the environment without treatment, polluting the water, air, and soil of the craft village [2,3]. Consequently, the steel and iron smelters are where dioxins are formed and released into the environment via stack gases and ashes [4,5,6]. It appears that environmental pollution studies in the DH craft village have not received much attention, particularly regarding sediment pollution, as no survey reports have been published. Surface water and industrial wastewater pollution exceeding the limits set by QCVN 08:2023/BTNMT and QCVN 40: 2011 has been documented for parameters such as BOD5, COD, coliform, TSS, total N, P, and heavy metals [7,8].

Dioxins represent a group of several hundred chemicals that fall into two closely related families: polychlorinated dibenzo-p-dioxins (PCDDs) and polychlorinated dibenzofurans (PCDFs). While these families encompass hundreds of different compounds, only certain ones are toxic, specifically those with chlorine atoms positioned in particular locations [9]. These organic compounds feature two benzene rings and can contain between one and eight chlorine atoms in their molecular structure. PCDD molecules include two oxygen atoms, whereas PCDF molecules have only one. Compounds of PCDD/F with at least four chlorine atoms at positions 2, 3, 7, 8 (2,3,7,8-substituted-PCDD/Fs) are toxic and have a high propensity for bioaccumulation in food chains, even spreading to humans [10,11,12]. PCDD/F compounds are white and exhibit stability in both alkaline and acidic environments. They are slightly soluble in water and resist evaporation but are readily soluble in fatty acids. These compounds are primarily found in sediment, soil, and suspended particles, and are adsorbed onto dust surfaces in the air [10]. Exposure to the most toxic isomers of dioxin and furan, such as 2,3,7,8-tetrachlorinated dibenzo-p-dioxin (2,3,7,8-TCDD) and 2,3,4,7,8-pentachlorinated dibenzofuran, is a known cause of cancer in both humans and mammals [13]. Dioxins are by-products in chemical manufacturing processes, including chlorophenol production, chlorobenzene synthesis, and pesticide manufacturing, as well as in other activities, such as paper manufacturing, recycling, non-ferrous metal smelting, iron smelting, scrap recycling, and solid waste incineration. Dioxin pollution in Vietnam is primarily known to originate from hotspots or the use of millions of gallons of “Agent Orange” during the Vietnam War from 1961 to 1971. Pollution from industrial activities, on the other hand, appears to receive less attention.

Sedimentary environments such as lakes, ponds, and basins serve as natural repositories for pollutants like dioxins, which originate from various human activities. These pollutants can accumulate in sediment over time and subsequently enter the food chain, posing significant risks to both human health and the surrounding ecosystem [14,15,16].

Although not naturally occurring compounds, dioxins/furans are found in many places around the world, formed from human industrial activities. In countries with long-standing industrial development, such as Europe and the United States, studies have also shown the presence of dioxins, causing sediment pollution, in rivers. For example, in Germany, dioxins were found in Elbe River sediments due to waste from a Manganese production plant upstream [17]; in Italy, dioxins were found in the Po River due to the influence of waste from industrial and residential areas [18]; in the United States, dioxins were found in sediment bioaccumulation on fish in the Tittabawassee and Saginaw Rivers, Michigan, USA [19]. Many countries have also detected dioxins/furans in the sediment of lakes and rivers. Table 1 shows some examples of dioxin concentration in some countries.

In Vietnam, the accumulation of dioxins in sediment has been documented in several key locations. For instance, coastal areas in Can Gio district and Ho Chi Minh City, as well as numerous lakes in Hanoi and Hue city, have shown notable levels of dioxin contamination [20]. Additionally, sediment samples collected from e-waste recycling sites in Bui Dau village, My Hao district, and Hung Yen province have revealed the presence of dioxin compounds [21]. Furthermore, sediment near the Bien Hoa airbase, a known hotspot for Agent Orange contamination, has also been found to be contaminated with dioxins [22]. The study of sediment in the Saigon River, the Mekong River, and coastal sediment near Vung Tau in southern Vietnam also revealed the presence of dioxins [23]. To address this issue, the Ministry of Natural Resources and Environment has implemented QCVN 43-2017/BTNMT, a national technical regulation on sediment quality. This regulation specifies a permissible limit for dioxin in freshwater sediment, set at 21.5 ng/kg TEQ of dry weight [24]. Such measures are crucial for mitigating the environmental and health risks associated with dioxin contamination in sedimentary environments.

**Table 1:** Examples of dioxin/furan concentration in lake and river sediments observed in previous studies.

No.	Country	River Name	Concentration (ng/kg d.w)	Ref.
1	Turkey	Yeşilırmak River	3.47–33.8 (TEQ)	[25]
2	China	Daliao River	0.28–29.01	[26]
	China	Dongting Lake	130–891 (TEQ)	[27]
3	Canada	Niagara river	160–620 (TEQ)	[28]
4	Italy	Venice Lagoon	0.5–2857	[29]
5	Hong Kong	Mai Po Marshes Nature Reserve	5000–6900	[30]
6	Germany	Elbe river	1500 (TEQ)	[17]
7	France	Rhone River	<1–73,020	[31]
8	USA	Saginaw River	57–46,500	[19]
		Shiawassee River	11–438	
9	Japan	Kankazi River	68–6100	[32]
10	Korea	Yeongsan river	1140	[33]
		Nakdong River	2940	
11	Vietnam	Cau River	321	[34]

The World Health Organization has established toxicity equivalence factors (TEFs) for dioxins to assess their toxicity and health risks to humans and mammals relative to the most toxic congener, 2,3,7,8-tetrachlorodibenzo-p-dioxin (TCDD), which is assigned a TEF of 1. Other dioxin congeners are assigned TEFs based on their relative toxicity compared to TCDD. For example, 1,2,3,7,8-pentachlorodibenzo-p-dioxin (PeCDD) has a TEF of 1, indicating that it is equally as toxic as TCDD [35]. The TEF values are used to calculate the toxic equivalent (TEQ) of a mixture of dioxins, with the TEQ representing the overall toxicity of the mixture based on the individual congeners and their respective TEFs. Early EPA documents recommended the use of the TEF approach for specific PCDDs and PCDFs for environmental risk assessment [36]. To capture the uncertainty in these assumptions, all TEFs were provided as order-of-magnitude estimates, and the EPA described their application as a “useful interim approach” [36]. A set of guiding criteria were developed for TEF approaches [35]. These criteria included the development of TEFs through scientific consensus. The assignment of global consensus TEFs for the DLCs, including the dioxin-like PCBs, has been reevaluated as new data have become available [37] and through the consensus judgment of expert panels (e.g., the WHO deliberations detailed [35]). Since then, TEF and TEQ have been widely used in research on dioxins and furans.

Previous studies on dioxin/furan are rare in Vietnam due to the lack of high-resolution gas chromatography coupled with high-resolution mass spectrometry equipment, which is mainly used to analyze dioxin/furan at present [38]. These studies mainly focus on areas with serious dioxin contamination in Vietnam, which are Agent Orange hot spots formed during the war, with little attention to dioxin pollution from human activities. To date, there has been no study on the occurrence of dioxins in Vietnamese craft villages. This initial study aims to investigate the occurrence and distribution of dioxins/furans in sediment samples collected from the effluent rivers of secondary steel recycling craft villages in Bac Ninh province, Vietnam. The samples will be analyzed for 17 isomers of dioxins/furans using HRGC/HRMS. The results will be evaluated and discussed regarding the concentration of these compounds, their distribution, and TEQ to demonstrate the level of their environmental hazard. This information will help policymakers to develop solutions to minimize the pollution caused by these highly toxic compounds.

## 2. Results

### 2.1. Occurrence of Dioxin/Furan Contaminants

The analysis results revealed a surprising and concerning finding that dioxins and furans were detected in all sediment samples in the DH area, with all 17/17 targeted isomers being present. The average total concentration of dioxins and furans in the samples was 288.86 ng/kg d.w. In terms of concentration, 2,3,7,8-TCDD had the smallest average concentration compared to other congeners in the dioxin group, with an average value of 0.578 ng/kg d.w and ranging from 0.193 to 1.131 ng/kg d.w. OCDD had the highest average concentration, at 145.4 ng/kg d.w, and ranged from 10.3–209.6 ng/kg d.w; subsequently, the second-highest concentration was found in 1,2,3,4,6,7,8-HpCDD at a concentration fluctuating from 1.47 ng/kg d.w to 20.87 ng/kg d.w. The concentration of the remaining dioxin compounds was determined to be in the range of 0.02–4.67 ng/kg d.w.

In the group of furan compounds, the lowest average concentration was 1.37 ng/kg d.w, belonging to 1,2,3,7,8,9-HxCDF rather than 2,3,7,8-TCDF, ranging from 0.112 to 2.186 ng/kg d.w. 1,2,3,4,6,7,8-HpCDF had the highest concentration among furan compounds in the range of 3.88–68.77 ng/kg d.w, with an average value of 34.98 ng/kg d.w; subsequently, the second-highest concentration was found in OCDF at a concentration fluctuating from 1.878 ng/kg d.w to 28.57 ng/kg d.w, with an average of 20.77 ng/kg d.w. Generally, slightly lower concentrations of furan compounds compared to dioxins were detected in the studied samples collected at the same positions in the secondary steel recycling village. Similar concentrations were found in 2,3,4,6,7,8-HxCDF (0.18–15.11 ng/kg d.w), 1,2,3,4,7,8-HxCDF (0.39–14.75 ng/kg d.w), and 1,2,3,6,7,8-HxCDF (0.34–14.54 ng/kg d.w), while low concentrations were determined in 1,2,3,4,7,8,9-HpCDF (0.28–5.23 ng/kg d.w) and 1,2,3,7,8,9-HxCDF (0.11–2.19 ng/kg d.w). Most furan compounds in the DH4 position had higher residues compared to the other sampling locations in Da Hoi secondary steel recycling village. The presence of Dioxin/Furan compounds in the sediment at Da Hoi is very concerning regarding the health of the local residents, as Da Hoi is very close to residential areas.

**Figure 1:**
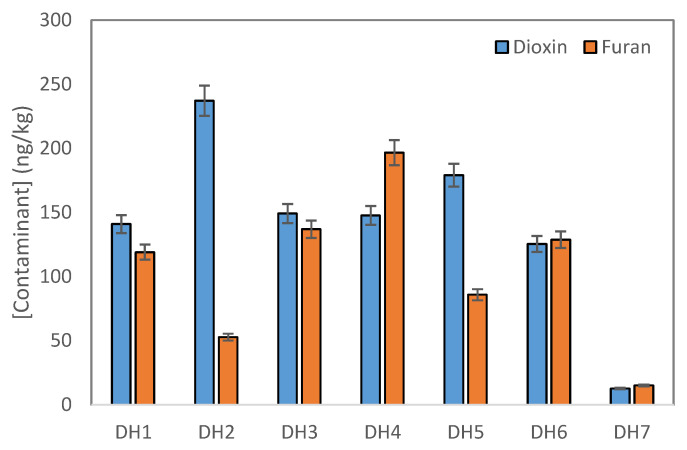
Concentration of total PCDDs and total PCDFs in sediment samples.

Figure 1 shows the total concentration of PCDDs and PCDFs in sediment samples at the Da Hoi secondary steel recycling village. A significant difference was found in the total concentration of PCDDs, as well as the total content of PCDFs, between the different sampling positions, and a significant difference was found between the total residue of PCDDs and those of PCDFs in each sample collecting position. The total residues of PCDDs ranged from 12.65 ng/kg d.w to 237.10 ng/kg d.w, while those of PCDFs were determined to have a value between 14.96 ng/kg d.w and 196.56 ng/kg d.w. In addition, the highest total concentration of PCDDs and PCDFs was reported to be 344.22 ng/kg d.w, in the DH4 position, followed by the DH2 position (289.76 ng/kg d.w), DH3 position (285.86 ng/kg d.w), DH5 position (264.74 ng/kg d.w), DH1 position (259.72 ng/kg d.w), and DH6 position (253.95 ng/kg d.w), with the lowest concentration was reported to be 27.61 ng/kg d.w, in the DH7 position. This indicates that the sample collected from a point along the river before it runs through the village had the lowest PCDD/Fs concentration. The samples collected downstream and from the canal running through the village show that the concentration of PCDD/Fs underwent a slight change and was from 8.5 to 12.5 times higher than the sample collected at the entrance of village. The dioxin levels in sample DH7 are quite low, indicating that this sample may not be related to the DH craft village area. Therefore, in our averaging calculations, we will exclude the value of this sample to reduce errors.

**Figure 2:**
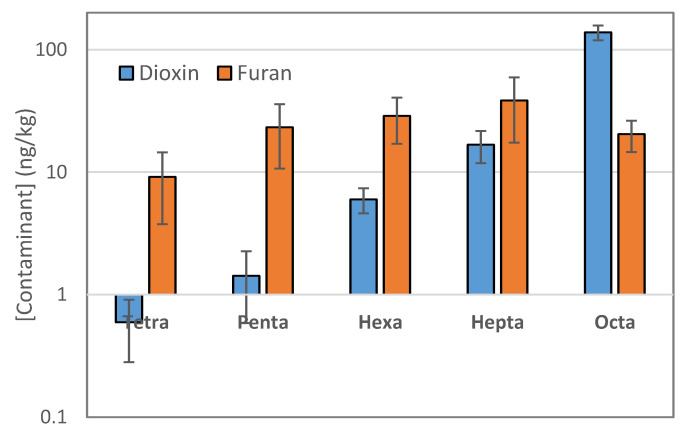
Concentration of dioxin by chlorine substitutes.

The levels of five groups of chlorine substitutes from tetra to octa are presented in Figure 2. The results show that furan has relatively consistent levels across the chlorine groups, with an average of 23.97 ng/kg d.w. In contrast, dioxin levels increase with an increase in the number of chlorine atoms in the molecule. We even observed that the dioxin levels increase proportionally with the number of chlorine atoms in the molecule according to the equation = 0.0021^1.3365 × chlorine^, with an R2 coefficient of up to 0.98. This phenomenon was also observed in some other research results of ours but has not been explained.

### 2.2. Distribution of Dioxins and Furans

As commented above, sample DH7 appears to be outside the waste discharge area of the Da Hoi craft village. Furthermore, due to the river flow direction from DH7 to DH3, the DH7 location is not affected by waste from the craft village, resulting in much lower levels of dioxin/furan being found at this location compared to other sampling points. The results suggest that the levels of dioxins and furans do not seem to depend on the flow direction because dioxins are not soluble in water and only accumulate in sediments.

In terms of total levels, the distribution of dioxins and furans is almost proportionally similar, except for samples DH2, DH4, and DH5. It seems that the closer the samples are to the production area, the higher the concentration of dioxins and furans. Among the dioxin compounds, the higher the number of chlorine atoms, the higher their percentage contribution to the total level (Figure 2). Thus, the percentage contribution is arranged in the order of Tetra < Penta < Hexa < Hepta < Octa. In contrast, the percentage distribution of furans shows a relatively consistent contribution, independent of the number of chlorine atoms (Figure 2).

**Figure 3:**
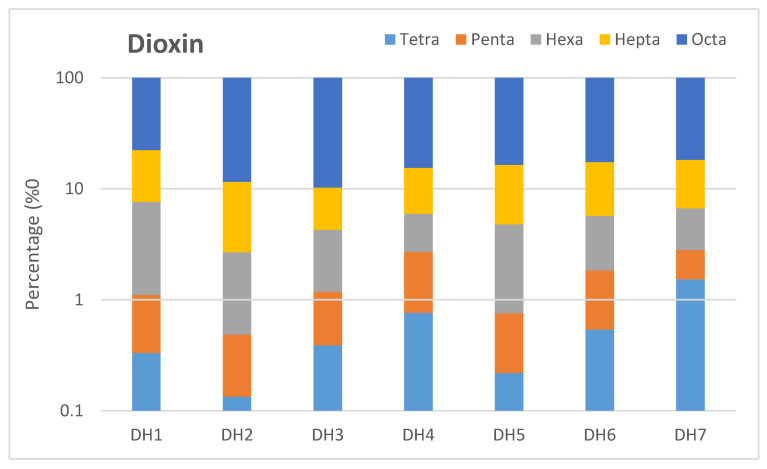
Distribution of different chlorine substituents groups of dioxin compounds.

The different proportions of dioxin compounds in the sediment samples collected at Da Hoi secondary steel recycling village are illustrated in Figure 3. The most dominant isomer was reported in OCDD, with a percentage ranging from 77.69% to 89.64%, followed by 1,2,3,4,6,7,8-HpCDD (5.98–14.66%), and 1,2,3,7,8,9-HxCDD (0.95–3.31%). Similar proportions were detected in the remaining dioxin compounds, including 1,2,3,7,8-PeCDD (0.35–1.92%), 1,2,3,6,7,8-HxCDD (0.75–1.87%), 1,2,3,4,7,8-HxCDD (0.49–1.36%), and 2,3,7,8-TCDD (0.13.1.53%), respectively.

**Figure 4:**
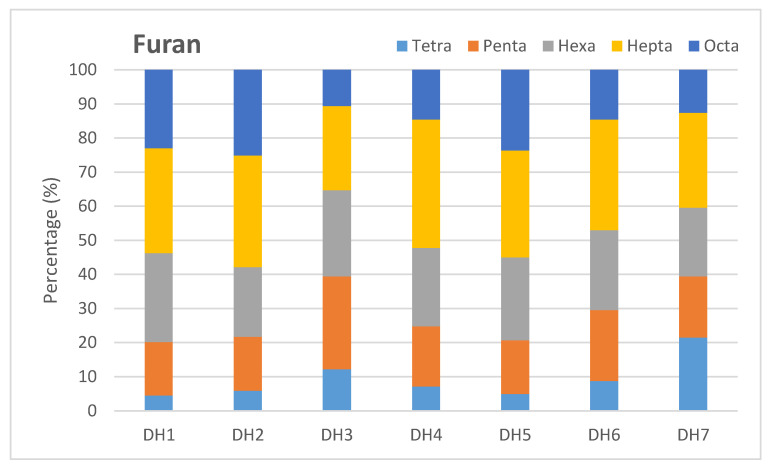
Distribution of different chlorine substituents groups of furan compounds.

In the case of furans, the highest ratio was found in 1,2,3,4,6,7,8-HpCDF (23.85–34.99%); the second and third positions belonged to OCDF (10.63–25.1%) and 2,3,7,8-TCDF (4.53–21.53%), respectively. Subsequently, the percentage of 1,2,3,7,8-PeCDF was determined at 5.65–17.27%, then 2,3,4,7,8-PeCDF (8.63–10.43%) and 1,2,3,4,7,8-HxCDF (7.40–10.40%), respectively. Similar percentages were reported in 1,2,3,6,7,8-HxCDF (6.55–8.89%) and 2,3,4,6,7,8-HxCDF (4.50–7.98%), while the lowest percentages were detected in 1,2,3,4,7,8,9-HpCDF (0.81–3.82%) and 1,2,3,7,8,9-HxCDF (0.46–1.84%), respectively (Figure 4).

**Figure 5:**
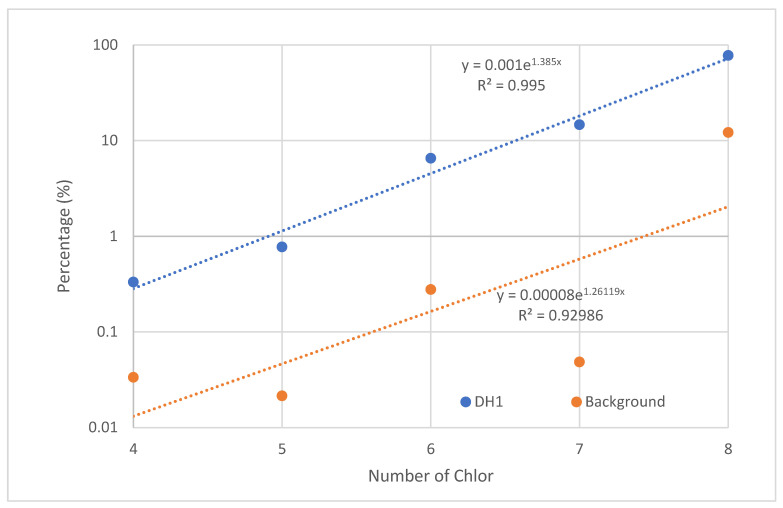
Increase in the number of chlorine substitutes related to the concentration.

An interesting finding is that we discovered a close relationship between the number of chlorine atoms and the concentration or percentage distribution of dioxins. Comparing the results of DH1, the curve shows an R2 very close to 1. We tested a sample taken from an area unrelated to industrial activities, but this sample did not show this correlation (Figure 5). The results of DH2–DH6 also show a similar correlation. However, DH7 shows a weaker correlation. Points DH1, DH2, and DH5 have the closest correlation, possibly due to direct dioxin waste being produced from steel recycling activities. Points DH3, DH4, and DH6 may have been partially contaminated from outside sources.

### 2.3. World Health Organization (WHO) TEQ for the PCDD/F

Although a comparison with QCVN 43:2017/BTNMT—National Technical Regulation on Sediment Quality shows that the total concentration of detected dioxins and furans is lower than the regulation of 21.5 ng/kg d.w TEQ, the presence of dioxins in the environment is still very concerning [24]. This is because the average toxicity of the samples is 10.93 ng/kg TEQ (excluding sample DH7), which is lower than QCVN 43:2017, but the range of 4.99–17.89 ng/kg d.w TEQ indicates that some samples are quite close to the limit.

**Figure 6:**
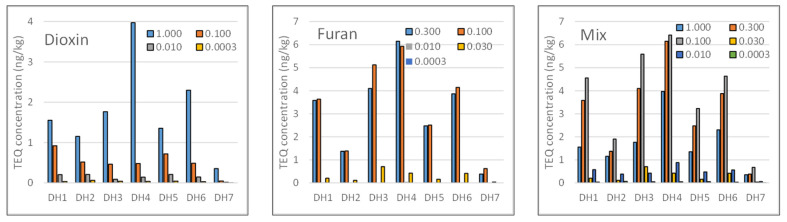
TEQ contribution of PCDD/Fs in sediment samples in the DH area. **Left***:* TEQ concentration of dioxins; **Center***:* TEQ concentration of furans; **Right***:* distribution by the coefficients of TEQ-WHO.

The total TEQ concentration of PCDD/F in the sediment samples collected at Da Hoi secondary steel recycling village is described in Table S3, with the following order: DH4 position (17.89 ng TEQ/kg d.w), then DH3 (12.63 ng TEQ/kg d.w) > DH6 (11.82 ng TEQ/kg d.w) > DH1 (10.50 ng TEQ/kg d.w) > DH5 (7.75 ng TEQ/kg d.w) > DH2 (4.99 ng TEQ/kg d.w) > DH7 (1.52 ng TEQ/kg d.w), respectively (Figure 6). It is interesting to note that the total TEQ content of PCDD/Fs in the samples collected upstream of the village was lower than all samples from the canal and river inside the village. This is concerning because, despite their very low concentrations, 2,3,7,8-TCDD and 1,2,3,7,8-PeCDD significantly contribute to the TEQ concentration. These are representatives of the tetra and penta groups. In contrast, OCDD has a very high concentration but contributes very little to the TEQ concentration.

The TEQ concentrations of furan that cause significant concern come from compounds with TEQ factors of 0.3 for 2,3,4,7,8-PeCDF and TEQ factors of 0.1 for compounds 2,3,7,8-TCDF, 1,2,3,4,7,8-HxCDF, 1,2,3,6,7,8-HxCDF, 1,2,3,7,8,9-HxCDF, and 2,3,4,6,7,8-HxCDF (Figure 6). These compounds represent the tetra, penta, and hexa groups.

**Figure 7:**
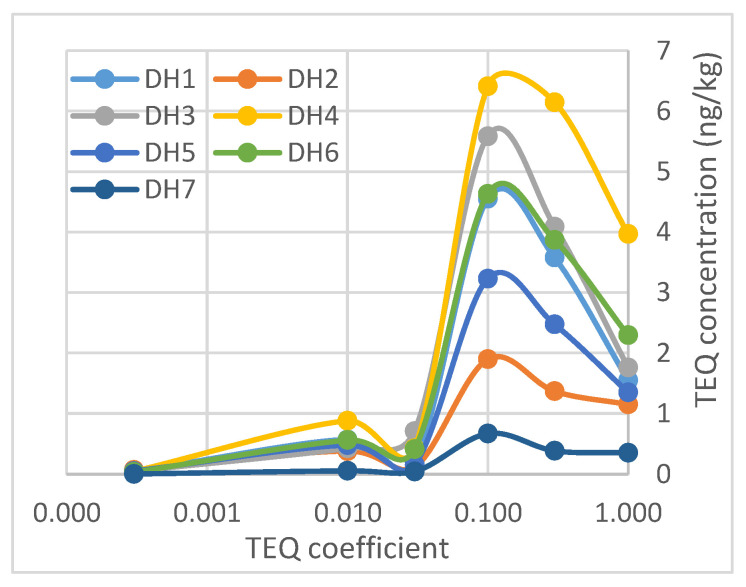
Correlation between TEQ coefficient and contribution of TEQ concentration.

The TEQ was used by the WHO to assess health risks to humans and mammals. Applying this method to the DH samples, the toxicities are found to be totally different. This result is unlike the concentration correlation discussed earlier. The order of decreasing toxicity of the samples can be arranged as follows: DH4 > DH3 > DH6 > DH1 > DH2 > DH5 > DH7 (Figure 7). This TEQ distribution is also unrelated to the flow direction of the river.

According to the previous studies, the TEQ concentration of dioxin compounds in the sediment samples collected at Bui Dau e-waste village in Hung Yen province, Vietnam, was 7.3 ng TEQ/kg d.w; at the West lake and Truc Bach lake in Ha Noi, the concentration was 9.6 ng TEQ/kg d.w; at the Cau river in Bac Giang province, the concentration was 0.75 ng TEQ/kg d.w [20,21,32,34]. This demonstrates that the TEQ concentrations of PCDD/Fs in sediment samples collected at Da Hoi secondary steel recycling village were higher than those of TEQ level of dioxin/furan compounds in sediment samples collected in other areas in Northern Vietnam. However, the TEQ contents of PCDD/Fs in the study samples were lower than those of sediment samples collected at the herbicides storage area in A Luoi airbases, middle Vietnam or Bien Hoa airbase in Southern Vietnam (91 ± 167 ng TEQ/kg d.w and 17–4860 ng TEQ/kg d.w) [22,39]. Particularly, OCDD accounts for the highest proportion, with a total concentration of 17 PCDD/Fs isomers in the sediment samples collected at Da Hoi secondary steel villages. This characteristic is similar to the sediment samples collected at some areas of Northern Vietnam, such as the West Lake and the Truc Bach Lake in Hanoi city, as well as Cau river in Bac Giang province and Can Gio mangrove forest in Southern Vietnam [20,34].

PCDD/Fs emitted from combustion processes, including steel sinter, showed intriguing characteristics regarding their composition. The total concentration of all 2,3,7,8-PCDD isomers was found to be lower than that of all 2,3,7,8-PCDF contents, with 2,3,4,7,8-PeCDF significantly contributing to the total TEQ concentration. The TEQ concentration profile of PCDD/Fs in soil and sediment samples from Agent Orange “hot-spots” in Vietnam, like Bien Hoa airbase and A Luoi airbase, revealed a dominance of 2,3,7,8-TCDD, accounting for from 42 to 99% of the total TEQ level [22,39,40].

Interestingly, natural sources of dioxins in sediment showed low concentrations of 2,3,7,8-PCDF and 2,3,7,8-TCDD, while PCDD content increased with the number of chlorine atoms in PCDD molecules. OCDD emerged as a strong leader in the distribution of all 17 2,3,7,8-PCDD/Fs congeners, comprising from 43 to 85% of the total PCDD/Fs [41].

The sediment samples from Da Hoi exhibited a unique profile, with OCDD dominating (36–72% of total PCDD/Fs) and PCDD levels increasing with the degree of chlorination. Surprisingly, the ratio of 2,3,7,8-TCDD was the lowest among all 17 PCDD/Fs isomers. In these samples, the TEQ distribution of 2,3,7,8-TCDD ranged from 4.4% to 12.7%, while 2,3,4,7,8-PeCDF accounted for 25.5–34.4% of the total TEQ, making it the most dominant compound.

Comparing PCDDs and PCDFs at different sites, such as DH1, DH2, DH3, and DH5, the total concentration of PCDDs exceeded that of PCDFs, whereas at DH4, DH6, and DH7, PCDFs were dominant. This suggests that the source of dioxin in sediment at DH4, DH6, and DH7 may be linked to secondary steel sinter, while at DH1, DH2, DH4, and DH5, pollution from various sources could be responsible.

## 3. Discussions

Da Hoi craft village has a history of development spanning hundreds of years and is the largest steel recycling hub in the country. The production activities in Da Hoi significantly contribute to the local and national economic growth. With over 1700 small-scale production facilities, Da Hoi has attracted a vast labor force [42]. However, due to its outdated production methods, Da Hoi has turned into an “environmental cancer hotspot”, not only poisoning the steelworkers but also spreading toxins to neighboring areas [43].

For a long time, Bac Ninh province has implemented various policies and technical solutions to control and mitigate environmental pollution from the activities of the Da Hoi craft village. These solutions may include relocating to industrial zones established in 2010 [43], annual environmental quality control [44], enhancing management measures and coordination across all levels [45], and innovating production technologies and improving environmental protection measures [45,46]. Some specific policies have been implemented, such as Resolution No. 02/2022/NQ-HDND and Decision No. 222/QD-UBND. Resolution No. 02 focuses on waste collection and transportation to reduce pollution spreading. Decision No. 222 will implement various investment projects, such as canal renovation. However, these measures seem to be superficial and fail to address the actual emissions of pollutants and pollution treatments. Moreover, the annual environmental pollution control only covers the conventional physical parameters specified in the QCVN system, such as BOD, COD, and ammonia [44], and is thus unable to detect more harmful substances like dioxins and furans.

Although the results of this study do not show a change in the concentration of dioxins/furans in the direction of the river flow, this is likely because the river has received waste at many different points from production activities. The presence of dioxins/furans in sediment is indeed an alarming sign for human health and the ecosystem. Although the solubility of dioxins/furans in water is very low, the potential for the bioaccumulation of dioxins/furans through the food chain/web is very high. This can also indirectly affect human health.

On the other hand, if river bottoms are disturbed, such as after experiencing heavy rain and flooding, these pollutants will be dispersed into the environment, directly affecting the health of the people. We know that people use water from this section of the river for the irrigation of flowers and crops. This will disperse dioxins/furans into the environment, which will then accumulate in agricultural products. Accordingly, dioxin/furan pollution will become more serious. On the other hand, the extraction of sand for construction in Vietnam is in great demand. We are not sure whether sand in Da Hoi village is being extracted for this purpose because these activities are often not disclosed. However, it will be very dangerous and cause widespread pollution if the sediment in this area is used for sand mining. Therefore, we recommend discontinuing all sand mining, water use, and fishing activities in this area.

Several solutions have been proposed to relocate the production activities of the village to industrial zones, but due to the policy of preserving the village’s operations, their implementation remains challenging. Furthermore, the profitable production continues day by day, making it seemingly impossible to halt operations to apply environmental protection measures. This study found alarming levels of dioxin/furan in the environment of Da Hoi; therefore, we propose some technical solutions for controlling and eliminating these pollutants. The specific proposed solutions include the following:

- Adding dioxin pollution parameters to the mandatory list for regular environmental pollution control in areas related to wastewater discharge from the production activities of the Da Hoi craft village. Although this study only monitored dioxin/furan in sediment, we recommend monitoring pollution in soil, water, air, and sediment because dioxin/furan seems to be present in all sample matrices.
- Early investment in building centralized solid and wastewater treatment systems in the Da Hoi craft village area, as the current waste collection and transportation methods may spread dioxin/furan pollutants to other areas. Furthermore, waste from steel recycling activities should be considered hazardous waste. Therefore, organizations collecting, transporting, and treating hazardous waste must be licensed.
- Issuing guidelines for steel recycling, especially regarding furnace temperature, because at low temperatures ranging from 450 to 800 °C, the formation of dioxin/furan is very high [47]. Therefore, the recommended temperature in steel recycling should be higher than 1200 °C to reduce the formation of these compounds. With the current dioxin/furan-contaminated sediment, detailed and extensive monitoring is required to accurately assess the level and load of pollution. Then, the dredging of mud and sediment around Da Hoi village can be carried out to dispose of dioxins as hazardous waste. This is a massive workload that requires further investment and time to implement.
- To protect the health of the people and prevent the widespread dispersion of pollutants, it is necessary to stop all sand mining activities, water extraction for domestic and agricultural use, as well as fishing and aquaculture in this area.

## 4. Materials and Methods

### 4.1. Chemicals

PCDD/Fs standards, including an internal standard spike solution (code: EDF-5999); a cleanup standard (code: EDF-6999); a labeled compound stock solution (EDF-8999); a precision and recovery standard solution (EDF-7999); and a calibration solutions (EDF-9999) were purchased from Cambridge Isotope Laboratories, USA. These standards were used to cause spikes in samples and create calibration curves. Solvents such as toluene, hexane, acetone, and nonane (purity > 99.9%), ordered from Merck, New Jersey, USA, were used to wash all tools, bottles, extracts, and clean up methods. The cleanup process involved the use of a multi-layer silica gel dioxin column (28397-U) and a dual-layer carbon reversible tube (28399-U) from Sigma-Aldrich, Steinheim, Germany. Nitrogen (purity > 99,999) from Messer Viet Nam was used for drying in this process.

### 4.2. Sampling

The Ekman bucket was used for sampling. One sampling point included a center sample point and four surrounding samples with approximately 1 m distance between them. Sediment samples were collected from a depth of 0-20 cm and were thoroughly mixed. Subsequently, gravel, wood chips, and plant residue were removed from samples. The samples were spread on stainless-steel tanks, then air-dried at room temperature and ground by a ceramic mortar. Finally, the sediment samples underwent a meticulous cleaning process, were placed in a dark glass container, labeled with all necessary information, and then stored in the freezer at −20 °C.

**Figure 8:**
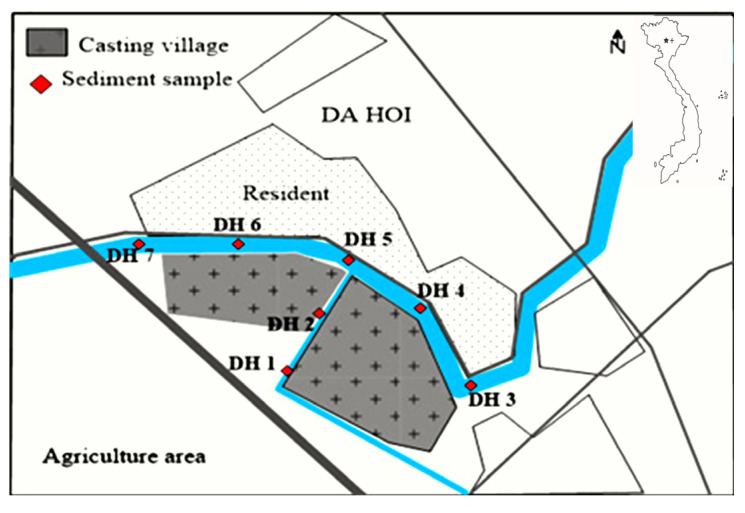
Sampling locations of sediment samples in the DH SSR area.

Samples were taken from seven locations in the DH SR area, including two samples, DH1 and DH2, in the canal, and five samples, DH3, DH4, DH5, DH6, and DH7, in the river along the village. The specific sampling locations are illustrated in Figure 8 and the coordinates of samples are shown in Table 2. Moreover, a quality control sample was supplied by IC10POP’s Ed 2022 proficiency testing, organized by LS Analytica, Italy. The accuracy of the result was evaluated by the Z-core method and values close to zero indicated high-accuracy results.

### 4.3. Sample Analysis

Approximate 5 g of a sample was weighted and mixed with a fixed amount of label standard containing 15 13C PCDD/F isotopes (200 pg 13C tetra to hepta-CDD/Fs and 13C 400pg Octa-CDD). Then, the mixture was extracted via the Soxhlet method using 300 mL DCM: hexane 1:4 for 16 h, cleaned by both the multi-layer silica gel dioxin column and dual-layer carbon reversible tube, and concentrated to about 1 mL by nitrogen gas. Finally, 50 µl of internal standard solution, comprising ^13^C-labelled 1,2,3,4-TCDD and 1,2,3,7,8,9-HxCDD at a concentration of 4 pg/µL, was added. The mixture was then concentrated to near dryness. Subsequently, 20 μL of nonane was added to dissolve the residue, which was then transferred into an insert vial for analysis. The samples were then analyzed by HRGC/HRMS DFS device (Thermo Fisher Scientific, Waltham, MA, USA). The TG-Dioxin chromatography column (Serial No. 1499302), which is similar to the DB5-MS column manufactured by Thermo Fisher Scientific company, with the following parameters: 60 m × 0.25 mm × 0.25 µm, was used for the isomer separation, coupled with High-Resolution Mass Spectrometry (HRMS) with a resolution of at least 10,000 and a source temperature set at 250 °C to detect the total ion chromatography (shown in Supplementary Material Figure S1).

Five calibration solutions containing the internal, label, and native 17 isomers of PCDD/Fs were used to establish a calibration curve. Meanwhile, 15 2,3,7,8-substituted PCDD/Fs isomers were obtained using isotope dilution (15 isomers) and 02 2,3,7,8-substituted PCDD/Fs isomers using the internal standard technique (1,2,3,7,8,9-HxCDD and OCDF) for quantitative determination [48].

### 4.4. Validation of Analytical Method

The analytical produce shown in Supplementary Material Figure S2. The method’s detection limits were determined based on a three-times signal-to-noise ratio, using blank samples spiked in accordance with the US EPA guidelines [49], ranging from 0.031 to 0.276 ng/kg for tetra to octa CDD/F (Supplementary Material Table S1). The sample processing efficiency of the method was evaluated based on the recovery efficiency of the 13C isotope standard, spiked during sample preparation processing, followed by the US EPA 1613b method [48]. The recovery efficiency of the label standard was in the range 34–81% (Supplementary Material Table S2). The method blank sample was prepared and analyzed to demonstrate the purification of the laboratory. In the analysis of blank samples, certain isomers were not detected, while others were identified at levels below the the method detection limit (Supplementary Material Table S3). This method was used to analyze sediment samples in the inter-laboratory testing program InterCinD IC10POP’s ed, 2022, organized by LS Analytica in 2022; the Z-core results of the 17 2,3,7,8-PCDD/Fs isomer concentration and the total dioxins TEQ content in sediment samples were in the range −1 < Z-cores < 1. The analysis process and Z-cores are shown in Supplementary Material Figure S3.

### 4.5. Calculation of TEQ

To calculate the TEQ for an environmental sample, the concentrations of individual congeners were first multiplied by their respective TEFs to produce congener-specific toxic equivalent concentrations (TEC). The individual TECs were then summed to obtain a total TEQ for the sample. The two equations used to calculate TEQs are shown in the box below.

(1) $${TEC}_{i}=C_{i}\times {TEF}_{i}$$

(2) $$TEQ=\sum_{i=1}^{n}({TEC}_{i})$$

where *C_i_* is the concentration of individual 2,3,7,8-PCDD/Fs isomers; *TEC_i_* is the toxic equivalent concentration of the *i* individual congener in the sample; *TEF_i_* is the individual toxic equivalent factor; *TEQ* is the toxic equivalent for the environmental sample; and *n* is the number of congeners that make up the *TEQ*.

Each dioxin or furan isomers have a TEF value, as provided in Table 3; meanwhile, 2,3,7,8-tetrachlorinated dibenzo-p-dioxin was the most toxic of the PCDDs group, with *TEF* = 1, and 2,3,4,7,8-pentachlorinated dibenzofuran is the highest TEF of the PCDFs group (TEF_2,3,4,7,8-PeCDF_ = 0.3). Another 2,3,7,8-PCDDs have a TEF ranging from 0.0003 to 1 and those of 2,3,7,8-PCDFs have a TEF ranging from 0.0003 to 0.3. The residue concentration of PCDD/Fs was converted to the total PCDD/Fs following the Van den Berg method [35].

Results were provided for individual congeners, as well as total dioxin and furan TEQs (TEQ_DF_), on a dry weight basis. The overall total dioxin TEQs (PCDDs and PCDFs) were then calculated as the sum of the TEQ_DF_. In cases where the concentrations of individual congeners were below the limit of reporting (LOR), the LOR was used in the TEQ calculation. This approach represents the upper limit of the TEQ, and is thus a conservative estimate.

## 5. Conclusions

This study provides the first evidence of the presence of dioxins/furans in sediment around the Da Hoi SSR craft village, with all 17 congeners detected in significant amounts. The average concentration was 288.86 ng/kg d.w. The smallest average concentration was observed for 2,3,7,8-TCDD, with an average value of 0.578 ng/kg d.w and a range from 0.193 to 1.131 ng/kg d.w. The highest concentration was found for OCDD, at 145.4 ng/kg d.w, and ranged from 10.3 to 209.6 ng/kg d.w. The TEQ levels of PCDD/F detected in the sediment samples ranged from 1.52 to 17.88 ng/kg TEQ, with an average value of 10.75 ng/kg TEQ. Although all TEQ values did not exceed the regulations of the Vietnamese Ministry of Natural Resources and Environment, the presence of dioxins/furans in all collected sediment samples is a cause for concern. The study determined that the levels of dioxins increased with the number of chlorine substitutions in the molecule, with the most toxicity attributed to the tetra and penta groups. The sample collected upstream before the village had TEQ levels of PCDD/Fs that were lower than those found in the canal and river inside the village. The TEQ concentration of PCDD/Fs in sediment in this area was higher than those at the lakes and rivers in Northern Vietnam, but lower than that in sediment samples from Agent Orange “hot-spots” in Vietnam. The source of dioxin in sediment in the studied area was not only secondary steel smelters but also other combustion processes. The distribution of dioxins/furans is concentrated in the production area but could potentially impact the health of residents living in close proximity. Further research is needed to accurately assess the pollution status of dioxins/furans and their impact on human health and the environment in order to develop appropriate mitigation measures.

## Figures and Tables

**Table 2:** Sampling positions of sediment in the Da Hoi steel recycling areas.

Code	N Coordinates	E Coordinates	Note
DH1	21°07′02″	105°55′16″	On the canal
DH2	21°07′11″	105°55′20″	On the canal
DH3	21°07′19″	105°55′03″	Before steel recycling area
DH4	21°07′18″	105°55′16″	Middle steel recycling area
DH5	21°07′15″	105°55′24″	Middle steel recycling area
DH6	21°07′03″	105°55′36″	Middle steel recycling area
DH7	21°06′56″	105°55′41″	After steel recycling area

**Table 3:** Recommended TEFs for human health risk assessment of dioxins and furans in this study.

No	Congener	TEF Value
1	2378-TCDD	1
2	12378-PeCDD	1
3	123478-HxCDD	0.1
4	123678-HxCDD	0.1
5	123789-HxCDD	0.1
6	1234678-HpCDD	0.01
7	OCDD	0.0003
8	2378-TCDF	0.1
9	12378-PeCDF	0.03
10	23478-PeCDF	0.3
11	123478-HxCDF	0.1
12	123678-HxCDF	0.1
13	123789-HxCDF	0.1
14	234678-HxCDF	0.1
15	1234678-HpCDF	0.01
16	1234789-HpCDF	0.01
17	OCDF	0.0003

## Data Availability

Data are contained within the article and Supplementary Materials can be shared.

## Supplementary Materials

The following supporting information can be downloaded at: https://www.mdpi.com/article/10.3390/molecules29081788/s1, Figure S1: The total ion chromatography of PCDD/Fs; Figure S2: Dioxins and furans analytical procedure by DFS system; Figure S3: The result of the inter-laboratory testing program InterCinD IC10POP’s ed 2022; Figure S4: High-resolution gas chromatography coupled with high-resolution mass spectrometry system Model DFS Thermo-USA; Figure S5:Final sample after processing transferred into an 150µl insert vial and placed in a 2ml dark vial; Figure S6: Sample after cleaning, removing impurities, transferred to a test tube for nitrogen gas blowing to remove all solvents, enriching the sample; Figure S7: The sample after passing through the multilayer column is impregnated onto an activated carbon column, then washed to clean and remove interfering impurities; Figure S8: The sample after extraction contains many impurities, therefore it needs to be cleaned by impregnating onto a multilayer carbon column, then washed to remove dirt and interfere with the dioxin signal; Figure S9: The sample after Soxhlet extraction may have a large volume, therefore it needs to reduce the sample volume by vacuum rotary evaporation to remove excess extraction solvent before cleaning through the chromatography column; Figure S10: The sample is placed into the Soxhlet extraction apparatus and extracted for 16 hours using a mixture of 300mL DCM:Hexane (1:4) to ensure complete separation of dioxin compounds from the sample; Figure S11: The sediment sample, approximately 100 (g), which has been cleaned and dried, is finely ground and sieved using the IKA MF 10 Basic grinding device; Figure S12: An Ekman grab was utilized for sediment sampling from the river; Table S1: Method detection limit of dioxins and furans in sediment; Table S2: The recovery efficiency (%)of 13C label compounds in blank and sediment samples; Table S3: The concentration of PCDD/Fs (ng/kg) in blank sample.

## Nomenclature

CVs	Craft villages
DH	Da Hoi
DLCs	Dioxin-Like Compounds
EPA	Environmental Protection Agency
HRGC/HRMS	High-Resolution Gas Chromatography/High-Resolution Mass Spectrometry
PCBs	Polychlorinated biphenyls
PCDDs	polychlorinated dibenzo-p-dioxins
PCDFs	polychlorinated dibenzofurans
PCDD/F	polychlorinated dibenzo-*p*-dioxin/polychlorinated dibenzofuran
TEFs	toxicity equivalence factors
TEQ	Toxic equivalent
SSR	Secondary Steel Recycling
WHO	World Health Organization
2, 3, 7, 8-PCDD	2,3,7,8-substituted-polychlorinated dibenzo-*p*-dioxin
2,3,7,8-TCDD	2,3,7,8-tetrachlorinated dibenzo-*p*-dioxin
1,2,3,7,8-PeCDD	1,2,3,7,8-pentachlorinated dibenzo-*p*-dioxin
1,2,3,4,7,8-HxCDD	1,2,3,4,7,8-hexachlorinated dibenzo-*p*-dioxin
1,2,3,6,7,8-HxCDD	1,2,3,6,7,8-hexachlorinated dibenzo-*p*-dioxin
1,2,3,7,8,9-HxCDD	1,2,3,7,8,9-hexachlorinated dibenzo-*p*-dioxin
1,2,3,4,6,7,8-HpCDD	1,2,3,4,6,7,8-heptachlorinated dibenzo-*p*-dioxin
OCDD	Octachlorinated dibenzo-*p*-dioxin
2, 3, 7, 8-PCDF	2,3,7,8-substituted-polychlorinated dibenzofuran
2,3,7,8-TCDF	2,3,7,8-tetrachlorinated dibenzofuran
1,2,3,7,8-PeCDF	1,2,3,7,8-pentachlorinated dibenzofuran
2,3,4,7,8-PeCDF	2,3,4,7,8-pentachlorinated dibenzofuran
1,2,3,4,7,8-HxCDF	1,2,3,4,7,8-hexachlorinated dibenzofuran
1,2,3,6,7,8-HxCDF	1,2,3,6,7,8-hexachlorinated dibenzofuran
1,2,3,7,8,9-HxCDF	1,2,3,7,8,9-hexachlorinated dibenzofuran
2,3,4,6,7,8-HxCDF	2,3,4,6,7,8-hexachlorinated dibenzofuran
1,2,3,4,6,7,8-HpCDF	1,2,3,4,6,7,8-heptachlorinated dibenzofuran
1,2,3,4,7,8,9-HpCDF	1,2,3,4,7,8,9-heptachlorinated dibenzofuran
OCDF	Octachlorinated dibenzofuran
