# Supplementary material for: A Comprehensive Evaluation of Dioxins and Furans Occurrence in River Sediments from a Secondary Steel Recycling Craft Village in Northern Vietnam
Source: Molecules. 2024 Apr 15;29(8):1788. doi: 10.3390/molecules29081788 (PMC11052440; doi:10.3390/molecules29081788)
Supplement: Supplementary file 1 [file molecules-29-01788-s001.zip › molecules-2935702-supplementary.pdf]

molecules

Supplementary materials

Comprehensive evaluation of dioxins and furans occurrence in river sediments from a Secondary Steel Recycling craft village in northern Vietnam

Hung Xuan Nguyen [1,2], Xuyen Thi Nguyen [1,2], Hang Thi Hong Mai [2,4], Huong Thi Nguyen [2], Nam Duc Vu [2], Thao Thi Phuong Pham [2], Trung Quang Nguyen [3], Dat Tien Nguyen [2], Nam Thanh Duong[2], Anh Le Tuan Hoang [2], Tung Ngoc Nguyen [2], Nhan Van Le [2], Ha Viet Dao [5], Minh Truong Ngoc [2], Minh Quang Bui [2,*]

1. Faculty of Chemistry, Graduate University of Science and Technology, Vietnam Academy of Science and Technology, 18 Hoang Quoc Viet Street, Cau Giay, Hanoi 11353, Vietnam
2. Center for High Technology Research and Development, Vietnam Academy of Science and Technology, 18 Hoang Quoc Viet Street, Cau Giay, Hanoi 11353, Vietnam
3. Institute of Environmental Science and Public Health, 18 Hoang Quoc Viet Street, Cau Giay, Hanoi 11353, Vietnam
4. Faculty of Chemistry, University of Natural Science, Hanoi National University, 19 Le Thanh Tong Street, Hoan Kiem, Hanoi, Vietnam
5. Institute of Oceanography, 1 Cau Da Street, Nha Trang, Khanh Hoa, Vietnam

* Correspondence: bui_quang_minh@yahoo.com; Tel.: (+84)-985173286

Citation: Nguyen, H.X.; Nguyen, X.T.; Mai, H.T.H.; Nguyen, H.T.; Vu, N.D.; Pham, T.T.P.; Nguyen, T.Q.; Nguyen, D.T.; Duong, N.T.; Le Tuan Hoang, A.; et al. Comprehensive evaluation of dioxins and furans occurrence in river sediments from a Secondary Steel Recycling craft village in northern Vietnam. *Molecules* **2024**, *29*, 1788. https://doi.org/10.3390/molecules29081788

Academic Editor(s): Name

Received: 12 March 2024
Revised: 7 April 2024
Accepted: 10 April 2024
Published: 15 April 2024

Copyright: © 2024 by the authors. Licensee MDPI, Basel, Switzerland. This article is an open access article distributed under the terms and conditions of the Creative Commons Attribution (CC BY) license (https://creativecommons.org/licenses/by/4.0/).

Contents

Table S1: Method detection limit of dioxins and furans in sediment

Compound	Method detection limit (ng/kg d.w)
2,3,7,8-TCDD	0.031
1,2,3,7,8-PeCDD	0.026
1,2,3,4,7,8-HxCDD	0.112
1,2,3,6,7,8-HxCDD	0.062
1,2,3,7,8,9-HxCDD	0.060
1,2,3,4,6,7,8-HpCDD	0.181
OCDD	0.193
2,3,7,8-TCDF	0.107
1,2,3,7,8-PeCDF	0.062
2,3,4,7,8-PeCDF	0.052
1,2,3,4,7,8-HxCDF	0.056
1,2,3,6,7,8-HxCDF	0.082
1,2,3,7,8,9-HxCDF	0.133
2,3,4,6,7,8-HxCDF	0.064
1,2,3,4,6,7,8-HpCDF	0.113
1,2,3,4,7,8,9-HpCDF	0.276
OCDF	0.199

Table S2: The recovery efficiency (%)of ^{13}C label compounds in blank and sediment samples

Compounds	DH 1	DH 2	DH 3	DH 4	DH 5	DH 6	DH 7	Blank
^{13}C-2,3,7,8-TCDD	64	54	49	61	60	42	60	67
^{13}C-2,3,7,8-TCDF	65	52	46	58	62	34	64	79
^{13}C-1,2,3,7,8-PeCDD	56	52	52	79	53	62	55	68
^{13}C-1,2,3,7,8-PeCDF	55	63	58	60	66	56	64	68
^{13}C-2,3,4,7,8-PeCDF	63	62	58	70	60	57	60	71
^{13}C-1,2,3,4,7,8-HxCDF	61	76	73	58	70	53	69	75
^{13}C-1,2,3,6,7,8-HxCDF	48	67	63	50	64	48	68	76
^{13}C-2,3,4,6,7,8-HxCDF	56	63	62	52	47	44	61	72
^{13}C-1,2,3,7,8,9-HxCDF	67	82	76	67	58	57	75	86
^{13}C-1,2,3,4,7,8-HxCDD	64	61	65	52	55	55	69	69
^{13}C-1,2,3,6,7,8-HxCDD	48	51	53	50	55	48	54	60
^{13}C-1,2,3,4,6,7,8-HpCDD	59	63	63	62	65	57	87	59
^{13}C-1,2,3,4,6,7,8-HpCDF	53	51	57	45	55	44	65	64
^{13}C-1,2,3,4,7,8,9-HpCDF	77	81	79	60	72	60	50	75
^{13}C-OCDD	70	61	58	61	76	52	61	41

Table S3: The concentration of PCDD/Fs (ng/kg) in blank sample

**Compounds**	**Blank sample**
2,3,7,8-TCDD	N.D.
1,2,3,7,8-PeCDD	0.008
1,2,3,4,7,8-HxCDD	N.D.
1,2,3,6,7,8-HxCDD	N.D.
1,2,3,7,8,9-HxCDD	N.D.
1,2,3,4,6,7,8-HpCDD	N.D.
OCDD	0.032
2,3,7,8-TCDF	0.038
1,2,3,7,8-PeCDF	0.024
2,3,4,7,8-PeCDF	N.D.
1,2,3,4,7,8-HxCDF	0.014
1,2,3,6,7,8-HxCDF	N.D.
1,2,3,7,8,9-HxCDF	0.006
2,3,4,6,7,8-HxCDF	N.D.
1,2,3,4,6,7,8-HpCDF	0.013
1,2,3,4,7,8,9-HpCDF	0.007
OCDF	0.079

N.D. = not detected

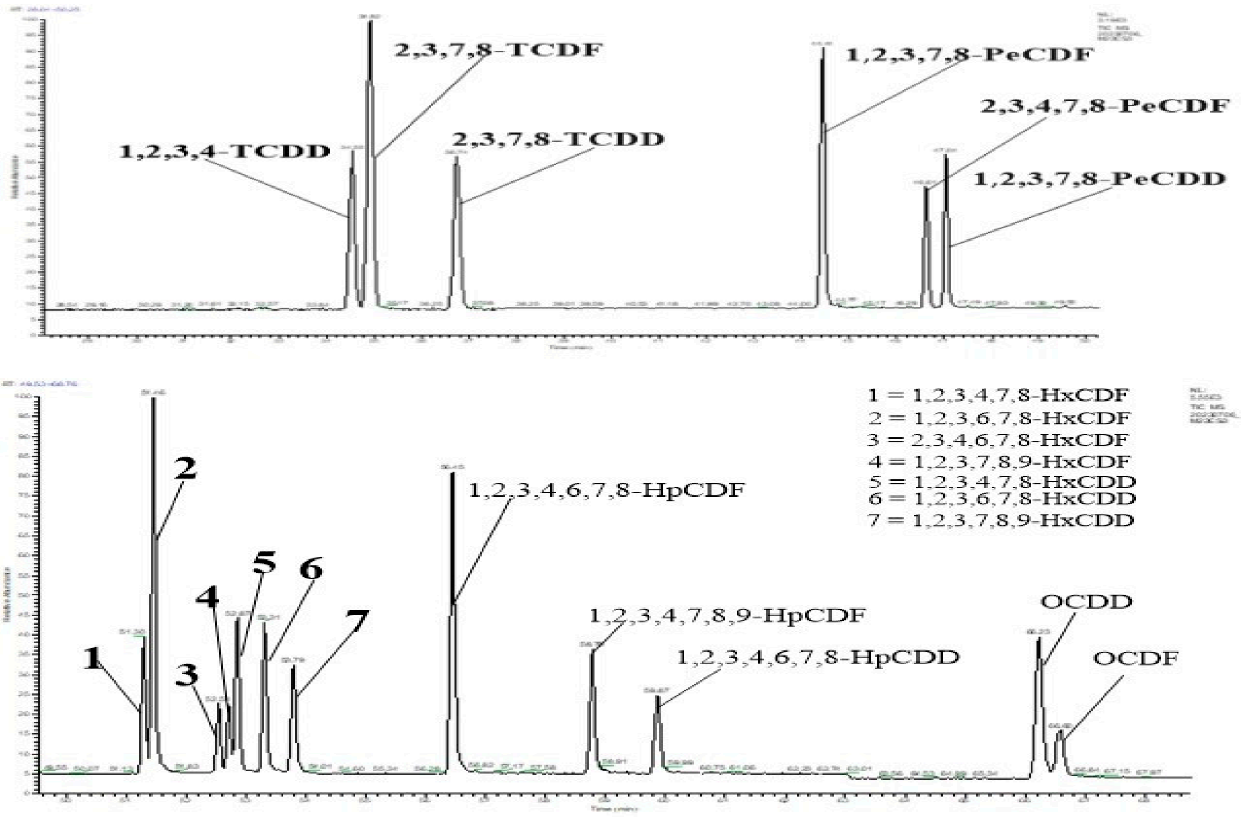

Figure S1: The total ion chromatography of PCDD/Fs

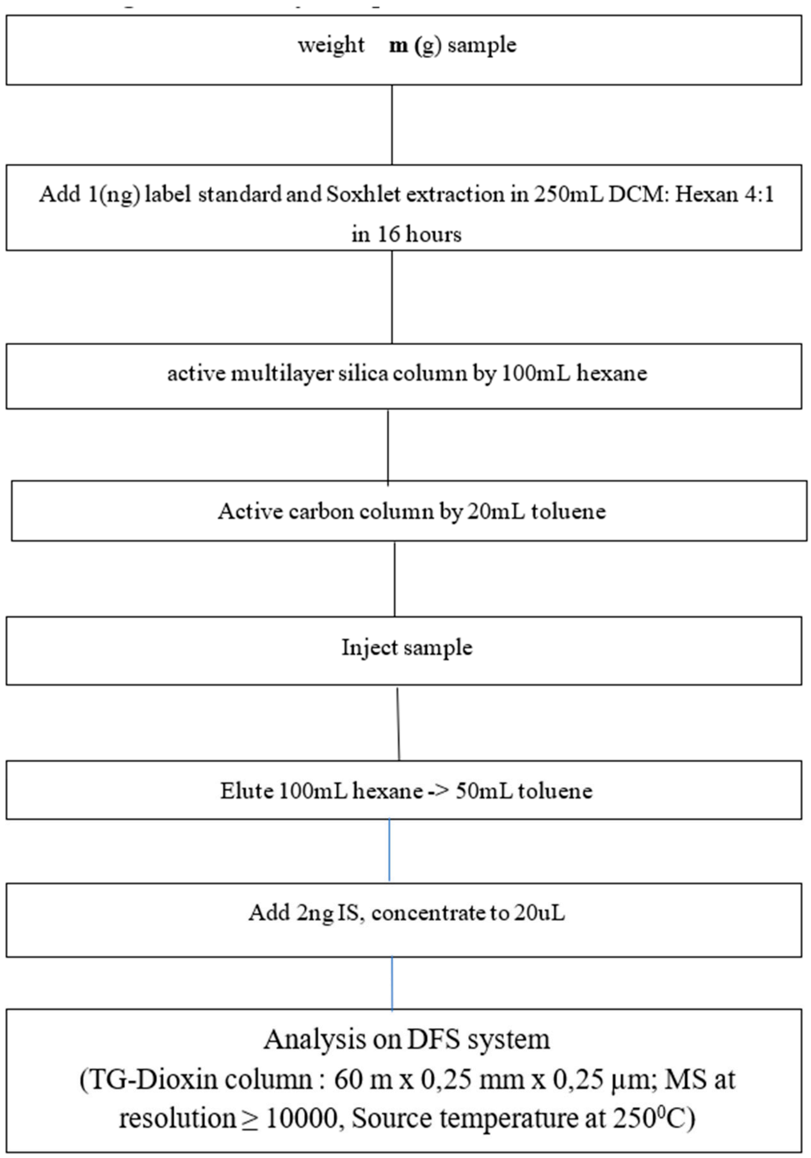

Figure S2. Dioxins and furans nalytical procedure by DFS system

MD20 Re[illegible]_Report_Rev00_10 January 2022

Z-scores - SEDIMENT

PCDD/F	Lab&replicate	[illegible]	[illegible]	[illegible]	[illegible]	Lab227-B	Lab227-C	Lab228-A	Lab228-B	Lab228-C
	GC/MS system	High	High	High	NA	NA	NA	High	High	High
2,3,7,8-TeCDD		-2.06	-2.63	-1.92	NA	NA	NA	0.22	0.66	0.79
1,2,3,7,8-PeCDD		-1.47	-1.56	-1.34	NA	NA	NA	0.48	0.28	-0.10
1,2,3,4,7,8-HxCDD		-1.57	-1.86	-1.57	NA	NA	NA	0.28	0.00	0.37
1,2,3,6,7,8-HxCDD		-1.57	-1.41	-1.25	NA	NA	NA	-0.38	-0.79	-0.79
1,2,3,7,8,9-HxCDD		-1.25	-1.33	-1.23	NA	NA	NA	-0.28	-0.42	-0.73
1,2,3,4,6,7,8-HpCDD		-1.56	-1.51	-1.65	NA	NA	NA	0.02	-0.43	0.65
OCDD		-1.26	-1.26	-1.31	NA	NA	NA	0.16	-0.16	0.95
2,3,7,8-TeCDF		-1.71	-1.76	-1.56	NA	NA	NA	0.66	0.00	0.01
1,2,3,7,8-PeCDF		-1.50	-1.52	-1.36	NA	NA	NA	-0.20	-0.43	-0.24
2,3,4,7,8-PeCDF		-0.48	-0.46	-0.28	NA	NA	NA	-0.36	-0.60	-0.63
1,2,3,4,7,8-HxCDF		-1.44	-1.52	-1.35	NA	NA	NA	-0.15	-0.30	0.07
1,2,3,6,7,8-HxCDF		-1.56	-1.51	-1.21	NA	NA	NA	0.36	-0.02	0.16
1,2,3,7,8,9-HxCDF		-0.35	-0.36	-0.25	NA	NA	NA	0.94	0.89	0.97
2,3,4,6,7,8-HxCDF		-0.98	-1.03	-0.94	NA	NA	NA	-0.30	-0.52	-0.49
1,2,3,4,6,7,8-HpCDF		-1.33	-1.36	-1.32	NA	NA	NA	0.02	-0.10	0.06
1,2,3,4,7,8,9-HpCDF		-1.42	-1.48	-1.49	NA	NA	NA	0.04	-0.02	0.07
OCDF		-1.14	-1.13	-1.14	NA	NA	NA	0.30	0.29	0.07
**TEQ (PCDD/F)**		-4.46	-4.58	-3.93	NA	NA	NA	0.17	-0.69	-0.20
**TEQ (PCDD/F) Upperbound (UpB)**		-4.46	-4.58	-3.93	NA	NA	NA	0.17	-0.69	-0.20

Figure S3: the result of the inter-laboratory testing program InterCinD IC10POP's ed 2022

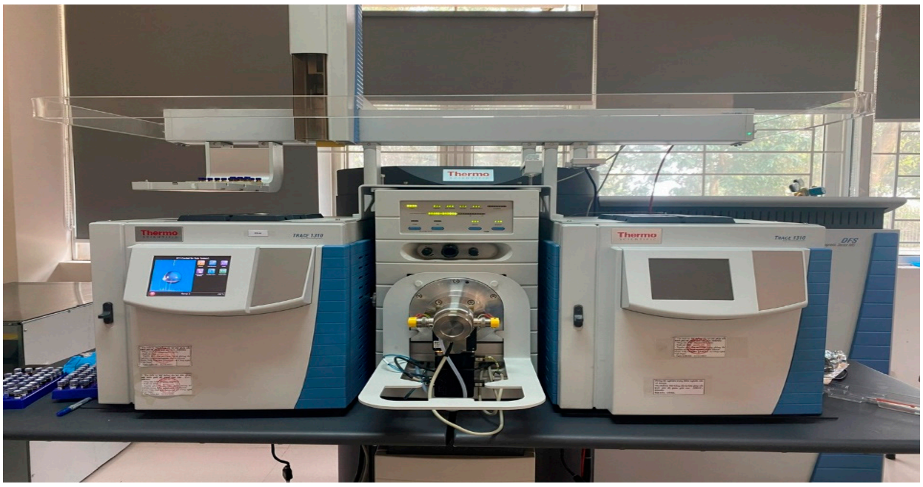

Figure S4. High-resolution gas chromatography coupled with high-resolution mass spectrometry system Model DFS Thermo-USA

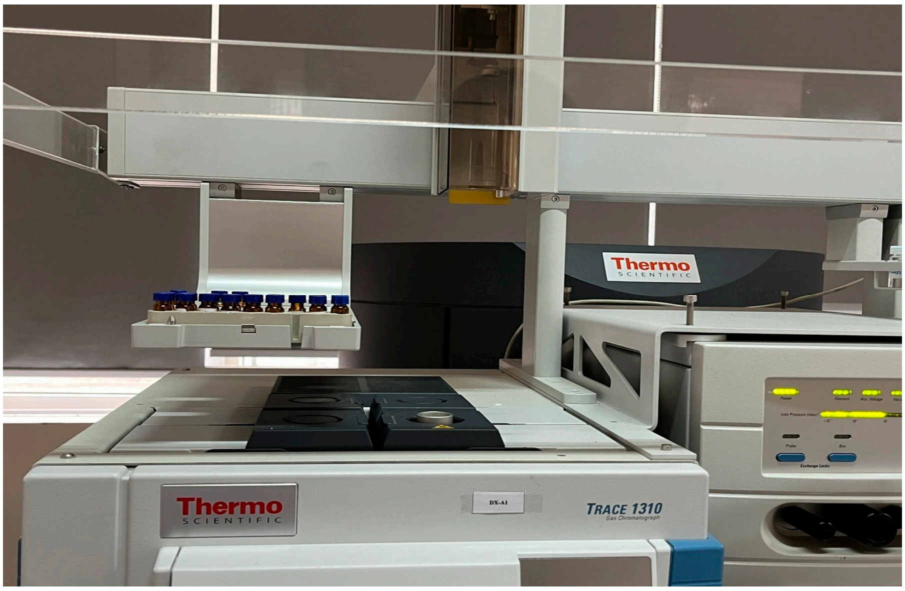

Figure S5. Final sample after processing transferred into an 150µl insert vial and placed in a 2ml dark vial

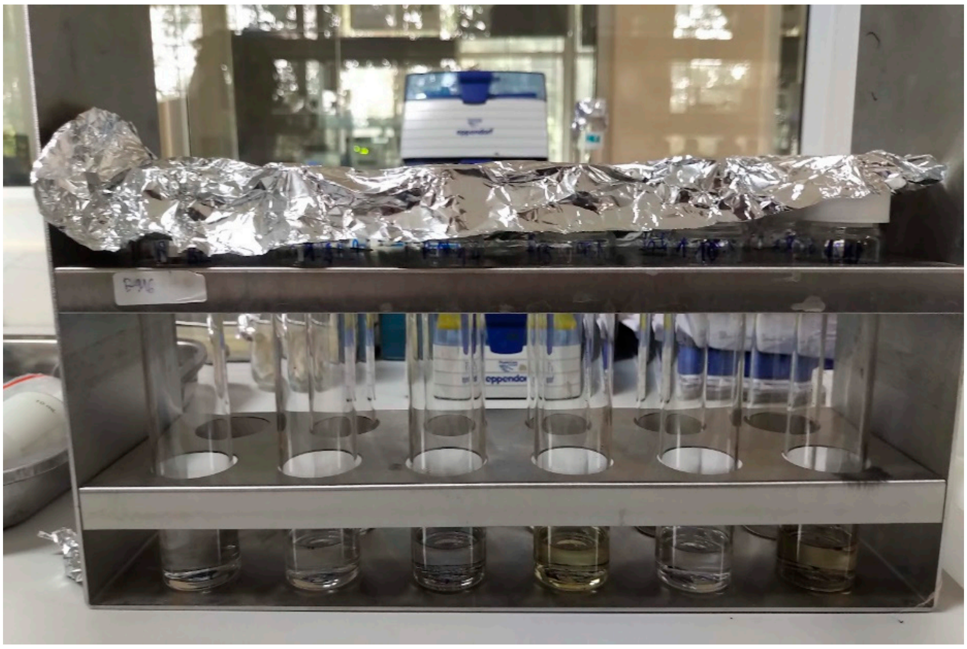

Figure S6: Sample after cleaning, removing impurities, transferred to a test tube for nitrogen gas blowing to remove all solvents, enriching the sample

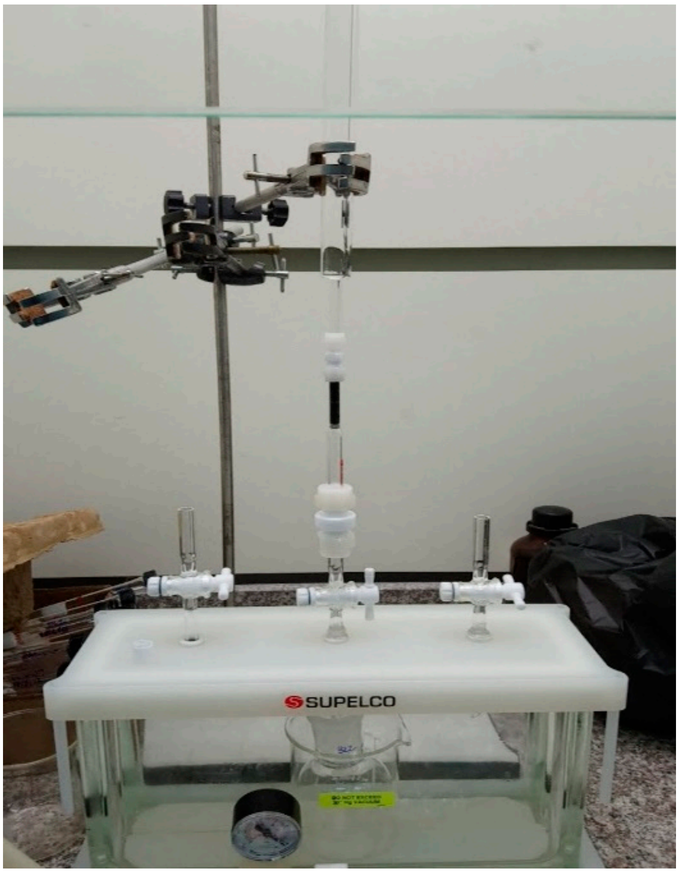

Figure S7: The sample after passing through the multilayer column is impregnated onto an activated carbon column, then washed to clean and remove interfering impurities.

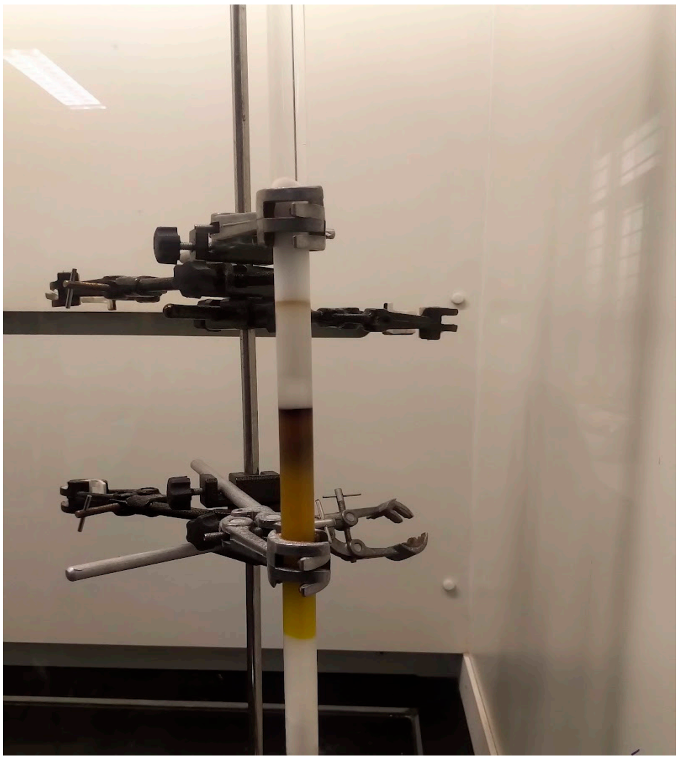

Figure S8: The sample after extraction contains many impurities, therefore it needs to be cleaned by impregnating onto a multilayer carbon column, then washed to remove dirt and interfere with the dioxin signal.

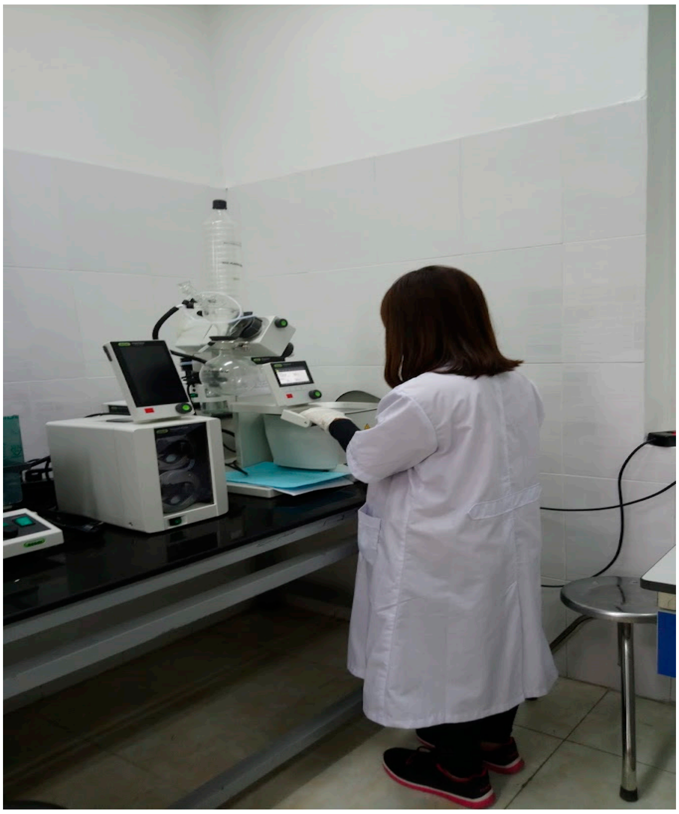

Figure S9: The sample after Soxhlet extraction may have a large volume, therefore it needs to reduce the sample volume by vacuum rotary evaporation to remove excess extraction solvent before cleaning through the chromatography column

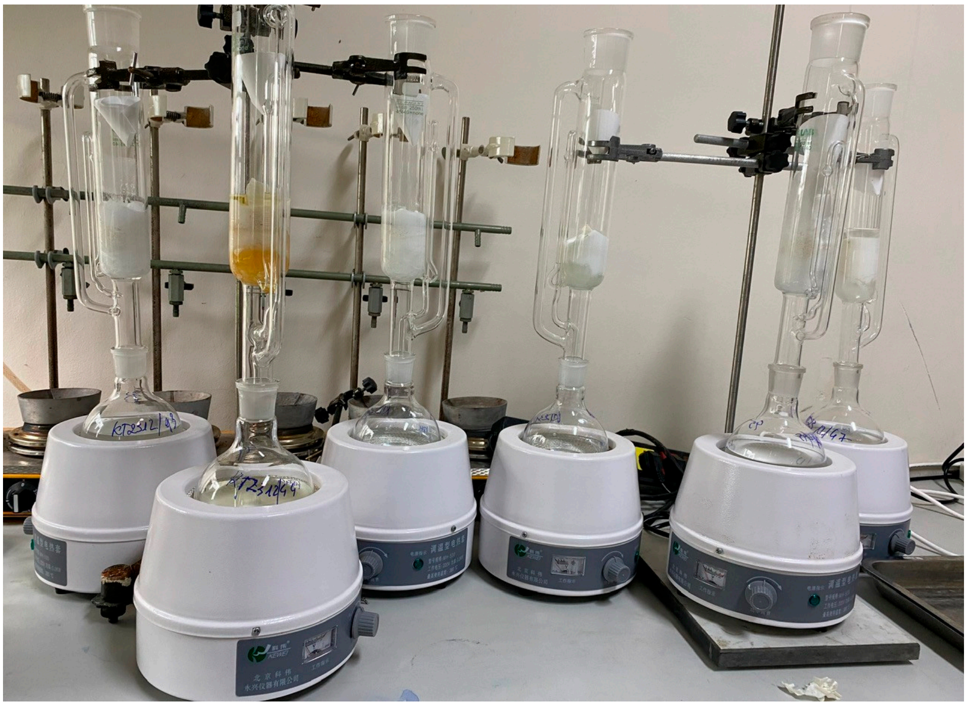

Figure S10: The sample is placed into the Soxhlet extraction apparatus and extracted for 16 hours using a mixture of 300mL DCM:Hexane (1:4) to ensure complete separation of dioxin compounds from the sample

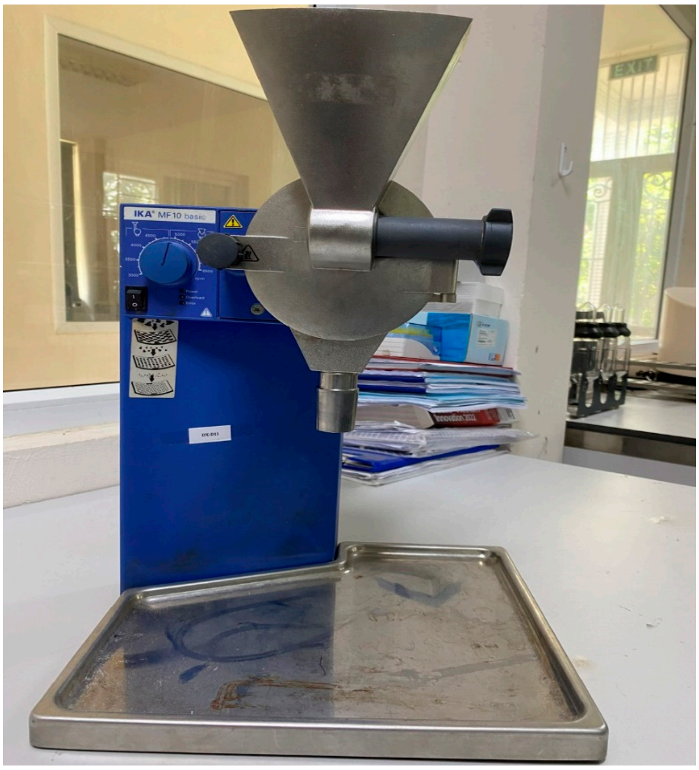

Figure S11: The sediment sample, approximately 100 (g), which has been cleaned and dried, is finely ground and sieved using the IKA MF 10 Basic grinding device

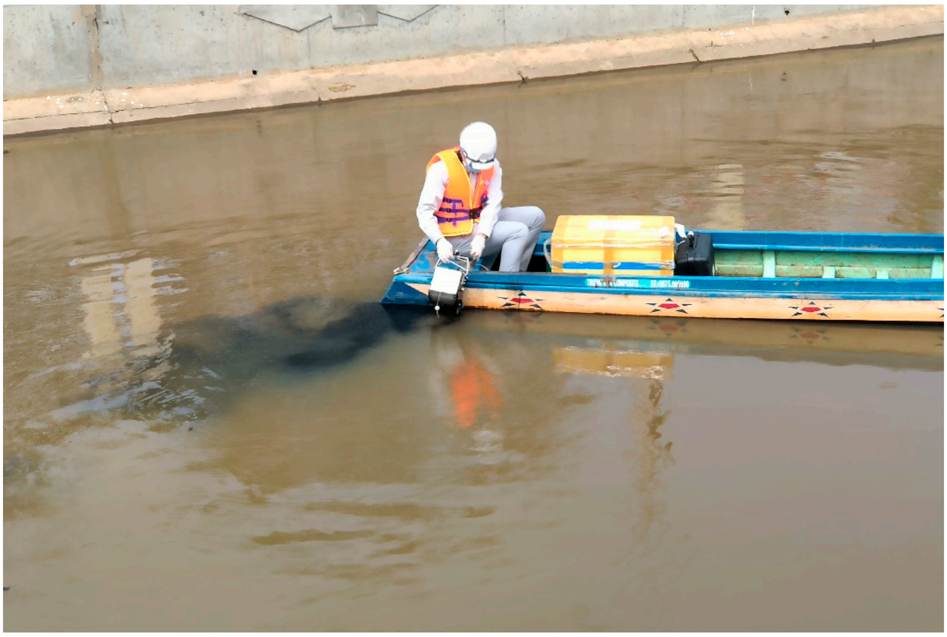

Figure S12: An Ekman grab was utilized for sediment sampling from the river

Disclaimer/Publisher's Note: The statements, opinions and data contained in all publications are solely those of the individual author(s) and contributor(s) and not of MDPI and/or the editor(s). MDPI and/or the editor(s) disclaim responsibility for any injury to people or property resulting from any ideas, methods, instructions or products referred to in the content.
